# Role of Cannabinoid Receptor Type 1 in Insulin Resistance and Its Biological Implications

**DOI:** 10.3390/ijms20092109

**Published:** 2019-04-29

**Authors:** Arulkumar Nagappan, Jooyeon Shin, Myeong Ho Jung

**Affiliations:** 1 Healthy Aging Korean Medical Research Center, School of Korean Medicine, Pusan National University, Yangsan 50612, Korea; arulbiotechtnau@gmail.com; 2 Division of Longevity and Biofunctional Medicine, School of Korean Medicine, Pusan National University, Yangsan 50612, Korea; jooyeonshine@naver.com

**Keywords:** cannabinoid receptor type 1, metabolic disorders, insulin resistance, obesity, diabetes

## Abstract

Endogenous cannabinoids (ECs) are lipid-signaling molecules that specifically bind to cannabinoid receptor types 1 and 2 (CB1R and CB2R) and are highly expressed in central and many peripheral tissues under pathological conditions. Activation of hepatic CB1R is associated with obesity, insulin resistance, and impaired metabolic function, owing to increased energy intake and storage, impaired glucose and lipid metabolism, and enhanced oxidative stress and inflammatory responses. Additionally, blocking peripheral CB1R improves insulin sensitivity and glucose metabolism and also reduces hepatic steatosis and body weight in obese mice. Thus, targeting EC receptors, especially CB1R, may provide a potential therapeutic strategy against obesity and insulin resistance. There are many CB1R antagonists, including inverse agonists and natural compounds that target CB1R and can reduce body weight, adiposity, and hepatic steatosis, and those that improve insulin sensitivity and reverse leptin resistance. Recently, the use of CB1R antagonists was suspended due to adverse central effects, and this caused a major setback in the development of CB1R antagonists. Recent studies, however, have focused on development of antagonists lacking adverse effects. In this review, we detail the important role of CB1R in hepatic insulin resistance and the possible underlying mechanisms, and the therapeutic potential of CB1R targeting is also discussed.

## 1. Introduction

Insulin resistance is a pathological condition characterized by the inability of insulin to regulate glucose and lipid metabolism in peripheral tissues even when insulin concentrations in the blood are elevated [1,2]. Insulin is essential for the regulation of glucose homeostasis and energy metabolism. Insulin resistance is a component of metabolic syndrome, which is associated with cardiovascular diseases and type 2 diabetes mellitus (T2DM) [1]. In particular, hepatic insulin resistance increases hepatic glucose production and triglyceride (TG) accumulation by impairing insulin-mediated inhibition of gluconeogenesis and by changing insulin-mediated TG metabolism, respectively, and these alterations contribute to hyperglycemia and dyslipidemia [1]. Additionally, obesity is a risk factor for insulin resistance and positively correlates with insulin resistance [3]. Therefore, management of hepatic insulin resistance and obesity provides an attractive strategy to combat T2DM and hepatic steatosis.

Endogenous cannabinoids (endocannabinoids, ECs) are lipid signaling molecules that regulate numerous biochemical processes, such as those involved in neuroprotection, pain, motor function, cardiovascular function, immune and inflammatory responses, energy balance, food intake, and cell proliferation [4,5]. The most widely studied ECs are arachidonoyl ethanolamide or anandamide and 2-arachidonoyl glycerol (2-AG), which bind to the specific receptors, cannabinoid receptor type 1 (CB1R) and CB2R, respectively. CB1R is found mainly in the brain, and CB2R is located in the cells of the immune system. Both CB1R and CB2R are also expressed in many peripheral tissues under pathological conditions [4,5]. Ample evidence exists suggesting that ECs play important roles in the regulation of metabolism [6,7]. CB1R is expressed in the liver, muscle, pancreas, and adipose tissue, which is highly involved in insulin action. EC signaling is deeply involved in insulin resistance and its related metabolic disorders.

**Figure 1:**
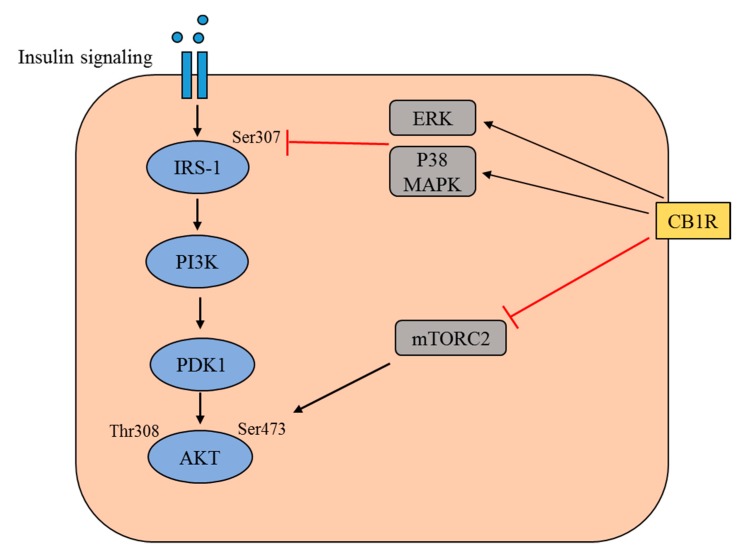
Schematic representation of the insulin signaling pathway and the modulation of this pathway by cannabinoid receptor type 1 (CB1R). The binding of insulin to the insulin receptor triggers a signaling cascade, which involves tyrosine phosphorylation of insulin receptor substrate 1 (IRS1) and the activation of phosphatidylinositol 3-kinase (PI3K). This leads to an increase in the level of phosphatidylinositol-3,4,5-trisphosphate, which recruits Akt to the plasma membrane alongside phosphatidylinositol-dependent kinase 1 (PDK1). In this cascade, Akt is phosphorylated at Thr308 by PDK1 and at Ser473 by mammalian target of rapamycin complex 2 (mTORC2). The activated CB1R mediates the activation of extracellular regulated kinase (ERK) and p38 mitogen-activated protein kinase (MAPK), which subsequently inhibits the Ser 307 phosphorylation of IRS1. Activated CB1R is also believed to inhibit the activation of mTORC2, thereby preventing the Ser473 phosphorylation of Akt.

The activation of CB1R in the liver is associated with obesity and metabolic complications such as insulin resistance and dyslipidemia by promoting the fatty acid uptake, lipogenesis, and adipogenesis [8]. Many studies have revealed that the activation of hepatic CB1R induces insulin resistance through several mechanisms [9,10,11] (Figure 1). Moreover, CB1R increases food intake by modulating the release of orexigenic and anorexigenic neuropeptides in hypothalamic neurons, thereby contributing to obesity [12,13,14,15]. Here, we discuss the importance of CB1R in hepatic insulin resistance, the possible underlying mechanisms, and the therapeutic potential of targeting CB1R.

## 2. Insulin Signaling Pathways

Insulin signal transduction is a complex mechanism regulated by numerous enzymes and modulatory proteins. The insulin receptor consists of two extracellular α subunits and two transmembrane β subunits, and binding of insulin to the receptor results in autophosphorylation on tyrosine residues and the subsequent tyrosine phosphorylation of insulin receptor substrates (IRS-1, IRS-2, and IRS-3) by the insulin receptor tyrosine kinase [16,17]. Receptor activation leads to phosphorylation of key tyrosine residues on IRSs that allows for association of IRSs with the regulatory subunit of phosphoinositide 3-kinase (PI3K) through its SRC homology 2 (SH2) domains (APS protein) [17]. Once activated, this protein creates suitable binding sites for IRSs that are then activated via phosphorylation by various insulin-induced kinases such as protein kinase C (PKC), serine/threonine-protein kinase 2 (SIK2), protein kinase B (AKT), p70 ribosomal protein S6 kinase 1 (S6K1), mammalian target of rapamycin (mTOR), extracellular signal-regulated kinase ½ (ERK1/2), and rho-associated coiled-coil-containing protein kinase 1 (ROCK1) [18]. Additionally, AMP-activated protein kinase (AMPK) and glycogen synthase kinase 3 (GSK3) are insulin-independent kinases that also phosphorylate IRSs and trigger downstream signal transduction [18].

Subsequently, the activated IRS-1 triggers signal transduction by binding to the catalytic subunit of PI3K, p110, and then phosphorylates phosphatidylinositol 4,5-bisphosphate (PIP2) to phosphatidylinositol 3,4,5-trisphosphate (PIP3) [19]. PIP3 is a potent effector of AKT, and active AKT inactivates GSK3, which is an inhibitor for glycogen synthase, thereby promoting glycogen synthesis [17]. Activation of AKT also requires the protein kinase 3-phosphoinositide-dependent protein kinase-1 (PDK1), which promotes the phosphorylation of AKT. The main function of insulin is to induce translocation of the glucose transporter-4 (GLUT-4), thereby stimulating glucose uptake into cells. Additionally, insulin activates non-IRS-dependent signaling pathways by other substrates such as heterotrimeric G proteins and the son of sevenless (SOS) growth factor [20].

## 3. Insulin Resistance

Insulin resistance is a pathological condition characterized by the inability of insulin to elicit a hormone response in insulin-dependent cells to regulate glucose and lipid metabolism [1,2]. Insulin resistance is inversely correlated with insulin sensitivity in insulin-dependent tissues [21]. There are multiple factors responsible for insulin resistance, including oxidative stress, inflammation, impairment of insulin receptors, endoplasmic reticulum stress, and mitochondrial dysfunction, and these factors trigger insulin resistance through various mechanisms in different type of tissues. It has been reported that protein-tyrosine phosphatase 1B (PTP1B), a non-transmembrane tyrosine phosphatase, acts as a negative regulator of insulin and leptin signaling. PTP1B can reverse insulin-induced phosphorylation in tyrosine residues of IRS-1, which results in impairment of insulin signal transduction [22]. It can also prevent weight gain and overfeeding by negative regulation of leptin signaling [23]. PTP1B knockout (PTP1B^−/−^) mice exhibit enhanced insulin sensitivity and tyrosine phosphorylation of IRS-1 in the liver and muscle tissues. Additionally, PTP1B^−/−^ mice are resistant to weight gain even on a high-fat diet. Moreover, PTP1B^−/−^ mice are hypersensitive to leptin, which is involved in the regulation of satiety and energy expenditure. Hence, PTP1B^−/−^ mice are lean and insulin-sensitive. The leptin signaling is also dependent on the tyrosine phosphorylation of IRS-1, which is mediated by the Janus kinase (JAK)-signal transducer and activator of transcription (STAT) pathway. Additionally, up-regulation of PTP1B has been reported in insulin resistance and obesity [24] and, thus, inhibition of PTP1B significantly ameliorated insulin resistance [23,25]. PTP1B-deficient animal model also possesses a lower risk of insulin resistance and obesity and exhibits improved insulin sensitivity in peripheral tissues [26].

Previous studies suggest that several cytokines and inflammatory mediators in the plasma are up-regulated in insulin resistance, including tumor necrosis factor-α (TNF-α), monocyte chemotactic protein-1 (MCP-1), C-reactive protein (CRP), and interleukin-6 (IL-6) [27]. TNF-α alone could disrupt insulin signaling via serine phosphorylation of IRS-1 and blocking of glucose entry into cells by reducing GLUT-4 in the skeletal muscle cells [28]. In addition, the c-Jun N-terminal kinase (JNK) pathway also stimulates IRS-1 serine phosphorylation in the mice liver, which results in insulin resistance [29]. Inhibition of the inhibitor of nuclear factor kappa-B kinase subunit beta (IKKβ) signaling pathway (a subunit of IκB kinase) also plays an important role in enhancing the insulin sensitivity of skeletal muscles, which is a key modulator in tissue inflammation [30]. The hypothesized mechanism linking inflammation and insulin resistance is the activation of IKKβ/ nuclear factor kappa light chain enhancer of activated B cells (NF-κB) and JNK pathways [29].

Oxidative stress can also contribute to insulin resistance by impairing insulin signal transduction [31]. In this context, oxidative stress activates IKKβ/NF-κB and JNK pathways, resulting in phosphorylation of IRS proteins and ultimately IRS degradation [32]. Additionally, interlinking pathways between oxidative stress and inflammation activate each other [33]. Oxidative stress also induces insulin resistance by blocking relocation of IRS-1 and PIP (phosphatidylinositol)-kinase, promoting phosphorylation of IRS-1, and decreasing GLUT-4 expression [34].

In addition to these factors, defects in serine phosphorylation of IRS-1 also plays a pivotal role in insulin resistance [35]. Impairment of IRS-1 activation may induce insulin resistance via dependent and independent phosphorylation of Akt [36]. Elements that reduce IRS-1 phosphorylation or activate serine IRS-1 phosphorylation at the 307 site cause defects in insulin signal transduction, ultimately resulting in insulin resistance [37,38]. Additionally, obesity increases free fatty acid (FFA) levels in the plasma that are harmful to most cells, and this lipotoxicity has been reported to enhance IRS-1 serine 307 phosphorylation. Yang et al. (2010) demonstrated that obesity-induced ER stress also plays a critical role in insulin resistance [39]. ER stress induces the expression of oxygen-regulated protein 150 (ORP150), a molecular chaperon protecting cells from ER stress, and this expression alters the phosphorylation of IRS-1, Akt, phosphoenolpyruvate carboxykinase (PEPCK), and glucose-6-phosphatase (G6Pase). These findings suggest that ER stress could play a pivotal role in insulin signaling and gluconeogenesis [40]. Apart from common factors that contribute to insulin resistance, activation of hepatic CB1R is also associated with obesity, insulin resistance, impaired glucose and lipid metabolism, and enhanced oxidative stress and inflammatory responses. Hence, it is important to determine the role of CB1R in obesity and insulin resistance.

## 4. Distribution and Biological Roles of CB1R in the Human Body

CB1R is found mainly in the central nervous system (CNS), particularly in several brain regions such as the olfactory bulb, hippocampus, basal ganglia, and cerebellum that show the highest CB1R expression [41]. The cerebral cortex, septum, amygdala, hypothalamus, parts of the brainstem, and the dorsal horn of the spinal cord manifest moderate CB1R expression, while the thalamus and the ventral horn of the spinal cord only weakly express CB1R. Cannabinoid type receptors are responsible for most of the psychoactive effects of cannabinoids. CB1R and the EC system are primarily involved in central neural phenomena and disorders, such as appetite and food intake, learning and memory, anxiety and depression, multiple sclerosis, neurodegeneration, and addiction [4,42,43,44]. Both CB1R agonists and antagonists influence feeding behavior in several in vivo mouse models. In fact, inhibition of CB1R results in reduced food intake in several different animal models [45,46,47]. Mutant mice (CB1^−/−^) exhibit a lean phenotype associated with reduced body weight and reduced fat mass [48]. Additionally, when anandamide is administered into the ventromedial nucleus of the rat hypothalamus, it stimulates appetite and induces overeating. However, pre-treatment with the CB1R antagonist (SR141716) impedes the anandamide-induced overeating [49]. Moreover, the early indication of Alzheimer’s disease pathogenesis is abundant expression of CB1R in the hippocampus and cerebral cortex region [50]. In brains affected by Alzheimer’s disease, the expression of microglial CB1R and CB2R is elevated, and neuronal CB1R expression, specifically in the hippocampus and basal ganglia, is reduced [51]. CB1R is also increased in the glial cells and has a critical role in the regulation of inflammatory cytokines [52].

Apart from the CNS, CB1R is highly expressed in the peripheral nervous system and various peripheral tissues [41,53,54,55]. Normally, the amount of CB1R is low in liver tissue [56]; however, under pathological conditions, the expression of CB1R in hepatic cells increases, thus contributing to hepatic insulin resistance, fibrosis, lipogenesis, and steatosis [53]. CB1R is also expressed in the cardiovascular system under pathological conditions, where it promotes disease progression and cardiac dysfunction [57]. Activation of CB1R leads to oxidative stress as well as inflammation and fibrosis affecting cardiomyocytes, vascular endothelial cells, and smooth muscle cells [58]. The activation of the CB1R in isolated rat atria results in feeble cardiomyocyte contractility [59]. Numerous in vivo studies have demonstrated that activation of CB1R promotes cardiomyocyte injury, oxidative stress, inflammation, and fibrosis, and augments collagen deposition and cardiomyocyte overgrowth [60,61,62]. Thus, chronic inhibition of CB1R with rimonabant improves cardiac functions in the early and late stages after myocardial infarction, decreases arterial stiffness, and reduces cardiac remodeling [63,64]. The expression of CB1R in the reproductive system has been shown to modulate oocyte maturation and embryonic development [65]. In particular, activation of CB1R drives oocyte maturation by modulating the phosphorylation status of Akt and ERK1/2 and enhancing embryo development [65]. Additionally, other studies have provided evidence supporting the involvement of CB1R in oocyte maturation [66,67]. It has also been demonstrated that CB1-KO mice are resistant to LPS-induced embryo resorption and exhibit a weaker uterine inflammatory response [68]. In the gastrointestinal (GI) tract, CB1R is located in the neuronal and non-neuronal cells of the intestinal mucosal region, including enteroendocrine cells, immune cells, and enterocytes [69]. CB1R can alter GI motility, secretion of gastric acid, fluids, neurotransmitters, hormones, and the permeability of the intestinal epithelium via the action of neuronal and non-neuronal cells [69]. Interestingly, 2-AG, an agonist of both CB1R and CB2R, has been shown to reduce colitis and related systemic and central inflammation [70]. Additionally, AM841, another potent agonist of these receptors, significantly reduces GI motility by activating CB1R in the small and large intestine [71]. Additionally, this compound can normalize GI transit under acute stress at an extremely low dose. The expression of CB1R has also been detected in adipose tissue, bone, skeletal muscle, reproductive cells, and various other types of cells [53]. The localization and functions of CB1R in the human body are detailed in Table 1.

## 5. Participation of CB1R in Obesity and Insulin Resistance

**Figure 2:**
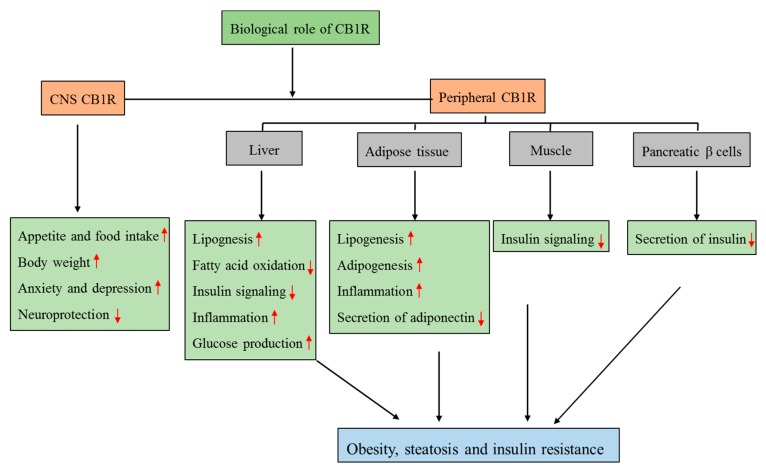
Biological role of CNS and peripheral CB1R in relation to obesity and insulin resistance. The localization of CB1R in the peripheral tissues such as liver, adipose tissue, muscle, and pancreatic β-cells has a potential role in obesity and related metabolic disorders without causing any adverse effects on the central nervous system. (Up arrow denotes increase and down arrow denotes decrease).

Evidence suggests that ECs are directly involved in the physiological control of food intake and energy utilization by targeting central and peripheral sites, including skeletal muscle, the liver, and adipose tissue [72]. CB1R activation in the CNS enhances food intake by regulating the activity of hypothalamic neurons and the subsequent release of orexigenic and anorexigenic neuropeptides [12]. Additionally, activation of CB1R signaling in adipose tissue and in the liver causes obesity and metabolic complications, including insulin resistance and dyslipidemia by increasing fatty acid uptake, lipogenesis, and adipogenesis [8] (Figure 2).

CB1R activation increases the activity of lipoprotein lipase (LPL), which promotes the hydrolysis of TGs into non-esterified fatty acids and their subsequent uptake by adipocytes, thus increasing fat storage in adipocytes [11,73]. CB1R also enhances fat storage via activation of lipogenic enzymes and inhibition of AMPK [73]. Additionally, previous studies have revealed that CB1R activation in white adipocytes stimulates adipogenesis, increases the amount of lipid droplets, and impairs biogenesis of mitochondria [74,75]. CB1R activation not only inhibits thermogenesis in brown adipocytes but also suppresses browning of white adipocytes to beige adipocytes [76]. Of note, CB1R-deficient mice possess a lean phenotype and manifest resistance to diet-induced changes [77]. Moreover, CB1R activation induces TNF-α expression and reduces the secretion of adiponectin [78]. Collectively, these findings suggest that CB1R activity in adipose tissue may contribute to obesity and insulin resistance via the regulation of white adipocyte expansion, brown adipocyte function, and inflammation. Evidence suggests that CB1R is expressed in human and murine skeletal muscle, and its activation has negative effects on insulin-mediated glucose uptake [79]. CB1R is also found in muscle mitochondria, and pharmacological blockade of CB1R regulates mitochondrial oxidative metabolism [80] and controls myoblast differentiation [81]. The activation of mitochondrial CB1 receptors in muscle cells has been reported to be associated with the mitochondrial regulation of oxidative activity [82]. Moreover, muscle CB1R ablation significantly improves whole-body performance and metabolism [83]. Previously, CB1R over-expression in β-cells was reported to enhance insulin release [84,85]. In contrast, recent evidence suggests that activation of CB1R induces apoptosis of pancreatic β-cells, revealing that it can directly inhibit insulin receptor activation by blocking insulin receptor kinase activity [86]. Coupled with this evidence, a lack of CB1R receptors in mice results in resistance to β-cell apoptosis, and improved insulin receptor activity and β-cell function [87,88]. These findings provide a cue that a crosslink exists between CB1R and insulin receptor signaling that influences β-cell survival and function.

**Figure 3:**
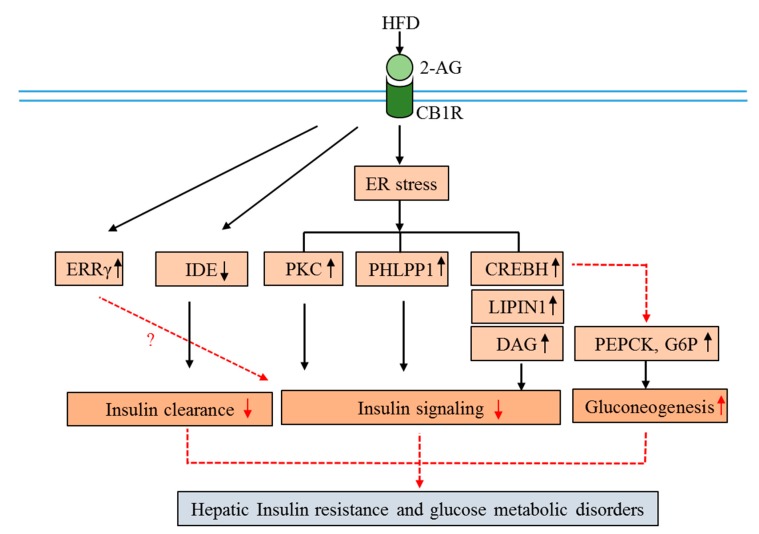
Molecular mechanisms of CB1R involvement in hepatic insulin resistance and glucose metabolism disorders. Down arrows indicate suppression, up arrows indicate activation, and dotted arrows indicate multiple pathways. (Abbreviations: CB1R, cannabinoid 1 receptor; 2-AG, 2-arachidonoylglycerol; HFD, high-fat diet; CREBH, cyclic AMP response element-binding protein H; ERRγ, estrogen-related receptors γ; PEPCK, phosphoenolpyruvate carboxykinase; G6Pase, glucose 6-phosphatase; IDE, insulin-degrading enzyme; PKC, protein kinase C; PHLPP1, S/T phosphatase PH domain and leucine-rich repeats protein phosphatase 1; DAG, diacylglycerol).

Insulin resistance is a pathological condition, in which the cells do not respond to the insulin hormone [1,2]. Both insulin resistance in peripheral tissues and relative deficiency in insulin secretion by β-cells are crucial for the development of T2DM [1]. As mentioned above, hepatic insulin resistance increases hepatic glucose production and TG accumulation, thereby causing hyperglycemia and dyslipidemia [1]. Hepatic insulin resistance is a general characteristic of most types of metabolic disorders such as obesity, dyslipidemia, metabolic syndrome, hypertension, nonalcoholic fatty liver disorder (NAFLD), and T2DM [2]. Hepatic CB1R activation induces insulin resistance via several mechanisms in an endoplasmic reticulum (ER) stress-dependent manner [9,10,11] (Figure 3). Engagement of CB1R inhibits insulin signaling by elevating inhibitory serine-307 phosphorylation of IRS1 and by stimulating the dephosphorylation of insulin-activated PKB/AKT through up-regulation of the S/T phosphatase PH domain and leucine-rich repeats protein phosphatase 1 (PHLPP1) [10]. Additionally, PHLPP1 directly dephosphorylates and inactivates Akt-2, which is a downstream target of CB1R that induces insulin resistance [89]. Activation of this receptor also stimulates the expression of lipin 1, a phosphatidic acid phosphatase, via an ER stress-inducible transcription factor called cAMP-responsive element-binding protein H (CREBH) [90]. This event subsequently leads to the accumulation of diacylglycerol (DAG), resulting in the phosphorylation of PKC and inhibition of insulin receptor signaling. Additionally, CB1R-mediated ER stress increases the production of ceramide by up-regulating de novo ceramide synthesis and suppressing ceramide degradation, ultimately inhibiting insulin signaling [91]. Aside from ER stress dependence, hepatic CB1R activation decreases insulin clearance as a consequence of down-regulation of insulin-degrading enzyme (IDE) [8]. High-fat diets (HFDs) induce hepatic insulin resistance in mice through CB1R-mediated inhibition of insulin signaling and clearance [10]. More recently, Liu et al. (2019) demonstrated that blockade of peripheral CB1R improves insulin sensitivity and glycemic control through the hepatic sirtuin 1 (Sirt1)/mTORC2/Akt signaling pathway, where it promotes energy expenditure by increasing fatty acid oxidation in a manner that is independent of hepatic Sirt1 and involves AMPK activation [92].

It has been demonstrated that CREBH, the target transcription factor of CB1R, in conjunction with insulin resistance, also plays a pivotal role in the regulation of hepatic gluconeogenesis during fasting as well as diet-induced insulin resistance in rodent models [93]. CB1R activation induces genes involved in gluconeogenesis, specifically PEPCK and G6Pase via CREBH, to elevate glucose production [93]. This evidence suggests that CREBH is the direct target of CB1R in the regulation of both hepatic insulin resistance and gluconeogenesis. Additionally, estrogen-related receptors (ERRs) substantially participate in CB1R-mediated insulin resistance and gluconeogenesis [94]. The ERR group has three members, α, β, and γ, that belong to the NR3B subfamily. It has been demonstrated that hepatic ERRγ is associated with the regulation of hepatic gluconeogenesis and is involved in the impairment of insulin signaling [95,96]. Moreover, ERRγ is a transcriptional mediator of the downstream effects of CB1R, where ERRγ blocks alcohol-induced cytochrome P450 2E1 (CYP2E1) expression, a major contributor to alcohol-induced reactive oxygen species (ROS) and liver injury, to ameliorate chronic alcohol-induced liver injury [94]. Accordingly, ERRγ is the downstream effector of CB1R and is associated with gluconeogenesis and insulin signaling. Therefore, ERRγ may play an important part in CB1R-mediated insulin resistance. Given this, further studies are necessary to validate the role of ERRγ in CB1R-mediated insulin resistance, and these findings will provide a new insight into the insulin resistance mechanism. Taken together, these data indicate that activation of CB1R under pathological conditions leads to obesity and contributes to insulin resistance. Finally, chronic high CB1R activity may progress to the development of T2DM.

The triggering of hepatic CB1R signaling is also involved in the development of hepatic steatosis as well as insulin resistance and T2DM [97]. Hepatic CB1R activation stimulates lipogenesis transcription factor and sterol-regulatory element-binding transcription factor 1c (SREBP1c) and increases accumulation of TGs via up-regulation of its downstream lipogenesis genes, including fatty acid synthase (FAS), stearoyl-coenzyme A (CoA) desaturase 1 (SCD1), and acetyl-CoA carboxylase (ACC), thus leading to hepatic steatosis. Additionally, CB1R activation decreases fatty acid oxidation via inhibition of AMPK and reduces the release of TG-rich very low-density lipoprotein (VLDL), which also contributes to hepatic steatosis [98,99,100]. Subsequently, hepatic TG accumulation causes insulin resistance. Consequently, activation of hepatic CB1R is involved in the development of hepatic steatosis, insulin resistance, and T2DM. In this regard, selective inhibition of hepatic CB1R signaling may be a molecular strategy for the treatment of T2DM and hepatic steatosis. Authors should discuss the results and their interpretation in relation to previous studies and the working hypotheses. The findings and their implications should be discussed in the broadest possible context. Future research directions may also be highlighted.

CB1R is known to form a heterodimer complex with class A G protein-coupled receptor (GPCR) superfamily, which includes human orexin 1 (OX1) and OX2 receptors [101]. Orexin receptor signaling triggers the production of 2-AG, which consequently activates the CB1R. CB1R has been reported to regulate many physiological functions such as analgesia, appetite, learning, and memory [42,102]. This suggests that the functional interaction (and heteromerization) between CB1R and OX1/OX2 receptor may contribute to the pathology of some diseases. There are several studies that have demonstrated the molecular and functional crosstalk between CB1R and OX1 receptor. This crosstalk may occur in the neuronal membrane of the nerve terminals at various brain regions. Specifically, the co-expression of CB1R and OX1 was observed in the hippocampus [103]. Hence, alteration in the expression of CB1R/OX1 may play a major role in CNS disease in certain brain regions. However, the interaction between the two receptors in vivo is still unclear.

## 6. The Functions of CB1R in Pancreatic β-Cells and Skeletal Muscle

Impaired insulin secretion by β-cells is a causal factor in the development of T2DM concomitant with insulin resistance. There are contradictory opinions regarding the synthesis of ECs in pancreatic islets, a process that varies from species to species. Few researchers claim that there is no synthesis of ECs in β-cells; however, CB1R expression has been shown in β-cells, of which activation enhances insulin release [84,85]. Recent findings revealed that ablation of CB1R in beta cells increases cell proliferation and early-phase insulin secretion, and also significantly prevents diet-induced intra-islet inflammation [88]. The CB1R antagonist ibipinabant mitigates β-cell loss without affecting body weight [104]. A recent study suggested that CB1R can suppress insulin signaling by blocking insulin receptor kinase activity via formation of a heteromeric complex with the insulin receptor [86]. The heteromeric-complex formation reduces phosphorylation of the pro-apoptotic regulator B-cell lymphoma 2-associated death promoter (BAD), thus leading to β-cell death [48]. It has been reported that insulin positively regulates β-cell proliferation in an autocrine manner through the insulin receptor signaling pathway. A recent report also suggested that CB1R and insulin receptor signaling function in the regulation of β-cell proliferation [87].

**Figure 4:**
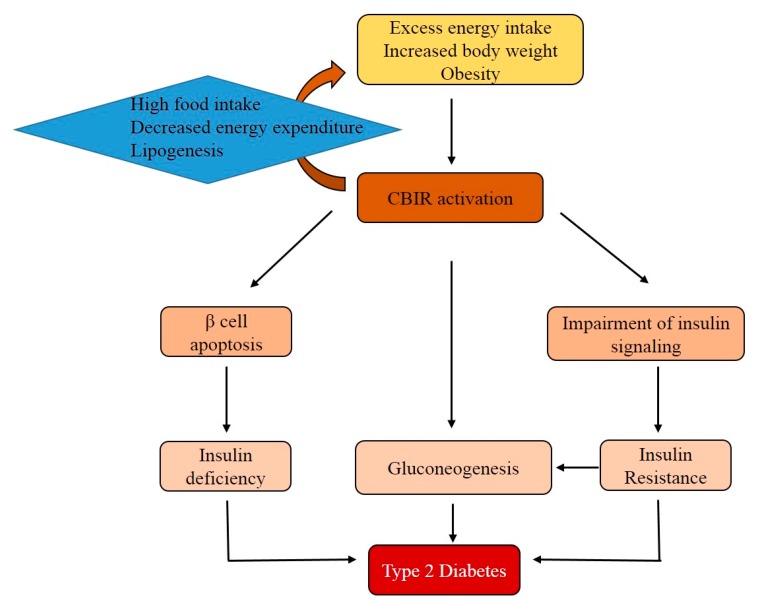
The role of the CB1R in the development of type 2 diabetes mellitus (T2DM). Excess food intake and obesity activates CB1R. The activation of CB1R is associated with gluconeogenesis and insulin resistance. Additionally, CB1R indirectly contributes to β-cell apoptosis. Finally, insulin resistance and relative insulin deficiency both contribute to the development of T2DM.

Additional evidence suggests that the CB1R activation in skeletal muscle decreases basal and insulin-mediated glucose uptake [79]. CB1R knockdown in muscle up-regulates insulin receptor signaling and regulates IL-6 and myostatin expression in a manner sufficient to regulate whole-body metabolism and physical performance [83]. The engagement of CB1R reduces the responsiveness of skeletal muscle to insulin by acting on the PI3K-PKB axis and the rapidly accelerated fibrosarcoma (Raf)-mitogen-activated protein kinase kinase (MEK) 1-2-ERK1/2 pathways [79]. Thus, CB1R signal transduction in peripheral tissues contributes to the development of T2DM through the induction of insulin resistance and impairment of insulin release (Figure 4).

## 7. The Therapeutic Potential

CB1R may be a promising therapeutic agent for obesity, insulin resistance, T2DM, and other metabolic syndromes. Rimonabant, an inverse agonist of central CB1R, has been recently approved as an antiobesity drug [105]. Specifically, this drug reduces body weight and insulin resistance and improves glucose metabolism. Its approval was suspended due to an increased risk of adverse psychiatric effects, such as anxiety, depression, and suicidal ideation. Since the withdrawal of rimonabant from the market, many investigators have studied peripheral CB1R inhibitors with milder side effects. A previous study suggests that the inhibition of CB1R by the selective antagonist SR141716 may provide an effective strategy for improving insulin sensitivity, and this approach promotes weight loss and insulin sensitivity by directly enhancing peripheral energy metabolism [106]. Additionally, SR141716 reduces 2-AG-induced insulin insensitivity in 3T3-L1 adipocytes [11]. A CB1R neutral antagonist AM6545 is equally effective as compared to its parent compound rimonabant at reversing hepatic steatosis but is less effective in reducing body weight, adiposity, insulin resistance, hyperleptinemia, and food intake [99]. A recent study indicates that the peripherally restricted CB1R inverse agonist JD5037 can reduce appetite, body weight, hepatic steatosis, and insulin resistance [107]. It exerts antiobesity effects by reversing leptin resistance. Another universal CB1R antagonist, ibipinabant, attenuates β-cell loss without changing the body weight [104].

Natural products derived from medicinal herbs are considered less toxic and may cause fewer adverse effects. N-oleoyl glycine, a lipoamino acid, promotes 3T3-L1 adipogenesis and insulin-mediated AKT signaling that is associated with the activation of CB1R [108]. More recently, we demonstrated that gomisin N (GN), a lignin derived from *Schisandra chinensis*, significantly improves hepatic CB1-mediated insulin resistance and gluconeogenesis [109]. GN reverses the 2-AG-mediated impairment of insulin signaling through modulation of insulin resistance-associated gene expression in HepG2 cells. Additionally, GN inhibits 2-AG-induced intracellular TG accumulation and glucose production in HepG2 cells by down-regulating genes associated with lipogenesis and gluconeogenesis, respectively. GN administration to HFD-fed obese mice reduces HFD-mediated inhibition of insulin signaling and increases fasting blood glucose and insulin levels. These findings indicate that GN protects against CB1-mediated impairment of hepatic insulin signaling and gluconeogenesis, thereby ameliorating hyperglycemia. Collectively, these data suggest that CB1R may be a therapeutic target in metabolic disorders associated with insulin resistance (Table 2). Moreover, CB1R blockade may be a beneficial outcome expected from the effects of antagonists undergoing toxicological screening and clinical testing. Natural compounds such as GN and N-oleoyl glycine require more attention, and their pharmacological effects need to be further investigated.

## 8. Conclusions

The role of CB1R in the EC system has recently received a great deal of attention due to its role as a master regulator of whole-body and cell metabolism and the observation that it is associated with critical processes modulating food intake, energy expenditure, lipogenesis, glucose uptake, insulin resistance, and gluconeogenesis. Under pathological conditions, an enhanced expression of CB1R is observed in the hepatic cells, which contributes to the hepatic insulin resistance, fibrosis, lipogenesis, and steatosis. The evidence strongly indicates that pharmacological blockade of CB1R provides a promising approach against obesity and metabolic syndrome. It should be noted that CB1R-deficient mice are resistant to obesity and metabolic syndrome, and CREBH is a target of CB1R in the regulation of hepatic insulin resistance. ERRγ is also a downstream effector of CB1R and may play an important part in CB1R-mediated insulin resistance. The role of CREBH and ERRγ in insulin resistance and obesity deserves special attention. Recently, the interaction between CB1R and orexin receptor was reported. It was demonstrated that the inhibition of peripheral CB1R improves insulin sensitivity and glycemic control through the hepatic Sirt1/mTORC2/Akt signaling pathway. Moreover, CB1R can suppress insulin signaling by blocking the insulin receptor kinase activity. The CB1R antagonist, ibipinabant attenuates β-cell loss without affecting the body weight. Additionally, CB1R exerts its antiobesity activity by reversing the leptin resistance. Further, CB1R reduces the insulin sensitivity of skeletal muscles by targeting the PI3K-PKB axis and the Raf-MEK1/2-ERK1/2 pathway. Natural compounds such as GN and N-oleoyl glycine that exert an inhibitory effect on CB1R deserve further research and clinical trials, as they may possess therapeutic potential. In this review, we discussed the important role and the possible underlying mechanisms of CB1R in hepatic insulin resistance and metabolic syndrome that will provide clues for the treatment of CB1R-mediated hepatic insulin resistance without causing any adverse effect on the CNS.

## Figures and Tables

**Table 1:** The distribution and functions of CB1R in the human body.

Sl.No.	Localization	Biological Role	Implication
1	Brain	Anxiety and depression, appetite and food intake, reward and addiction, neuroprotection, neural development, and sleep	CB1R overexpression results in increased food intake and induces overeating.
2	Liver	Liver steatosis, fibrosis, insulin resistance, lipogenesis, splanchnic, and vasodilation	CB1R overexpression induces insulin resistance and obesity.
3	Cardiovascular system	Cardiac function, negative inotropy, and vasodilation	CB1R overexpression promotes cardiomyocyte injury, oxidative stress, inflammation, and fibrosis.
4	Skeletal muscle	Energy metabolism and muscle fiber formation	CB1R overexpression impairs insulin signaling.
5	Reproductive system	Fertility regulation, embryo implantation, and embryonic development	Activation of CB1R modulates the oocyte maturation and embryonic development.
6	GI tract	GI motility, enteroendocrine function, and energy balance.	CB1R agonist reduces GI motility and colitis-related inflammation.

**Table 2:** The list of CB1R-specific antagonists, inverse agonists, and compounds that improve insulin resistance.

Sl.No.	Compounds	Biological Benefits	Cell Models
1	SR141716	Antiobesity, hepatoprotective, promoted insulin sensitivity, reduced aging-related insulin resistance, and reduced metabolic dysfunction	Mouse 3T3 F442A cells, obese (fa/fa) rats, 3T3-L1 adipocytes, rat L6 myotubes, and C57BL/6 mice
2	AM6545	Cardiometabolic risk	HFD-fed obese mice and CB1R^−/−^ transgenic mice
3	JD5037	Antiobesity effects by reversing leptin resistance	Diet-induced obesity (DIO) mice
4	Ibipinabant	Antidiabetic effects	β-cells
5	Gomisin N	Reduced insulin resistance and gluconeogenesis	HepG2 cells
6	N-oleoyl glycine	Reduced adipogenesis and increased insulin sensitivity	3T3-L1 adipocytes
